# Association of protein function-altering variants with cardiometabolic traits: the strong heart study

**DOI:** 10.1038/s41598-022-12866-2

**Published:** 2022-06-04

**Authors:** Yue Shan, Shelley A. Cole, Karin Haack, Phillip E. Melton, Lyle G. Best, Christopher Bizon, Sayuko Kobes, Çiğdem Köroğlu, Leslie J. Baier, Robert L. Hanson, Serena Sanna, Yun Li, Nora Franceschini

**Affiliations:** 1 Department of Biostatistics, University of North Carolina, Chapel Hill, NC USA; 2 Texas Biomedical Research Institute, San Antonio, TX USA; 3 The Curtin UWA Centre for Genetic Origins of Health and Disease, Faculty of Health Sciences, Curtin University and Faculty of Health and Medical Sciences, The University of Western Australia, Crawley, WA Australia; 4 School of Pharmacy and Biomedical Sciences, Faculty of Health Sciences, Curtin University, Bentley, WA Australia; 5 Menzies Medical Research Institute, University of Tasmania, Hobart, TAS Australia; 6 Missouri Breaks Industries Research Inc, Eagle Butte, SD USA; 7 Renaissance Computing Institute, University of North Carolina, Chapel Hill, NC USA; 8 Phoenix Epidemiology and Clinical Research Branch, NIDDK, NIH, Bethesda, USA; 9 Department of Genetics, University of Groningen, University Medical Center Groningen, Groningen, The Netherlands; 10 Istituto Di Ricerca Genetica E Biomedica (IRGB), Consiglio Nazionale Delle Ricerche (CNR), Monserrato, Italy; 11 Departments of Genetics and Computer Science, University of North Carolina, Chapel Hill, NC USA; 12 Department of Epidemiology, University of North Carolina, Chapel Hill, NC USA; 13 Gillings School of Global Public Health, University of North Carolina, Chapel Hill, NC USA

**Keywords:** Genetics, Diseases, Endocrinology, Pathogenesis, Risk factors

## Abstract

Clinical and biomarker phenotypic associations for carriers of protein function-altering variants may help to elucidate gene function and health effects in populations. We genotyped 1127 Strong Heart Family Study participants for protein function-altering single nucleotide variants (SNV) and indels selected from a low coverage whole exome sequencing of American Indians. We tested the association of each SNV/indel with 35 cardiometabolic traits. Among 1206 variants (average minor allele count = 20, range of 1 to 1064), ~ 43% were not present in publicly available repositories. We identified seven SNV-trait significant associations including a missense SNV at *ABCA10* (rs779392624, *p* = 8 × 10^–9^) associated with fasting triglycerides, which gene product is involved in macrophage lipid homeostasis. Among non-diabetic individuals, missense SNVs at four genes were associated with fasting insulin adjusted for BMI (*PHIL,* chr6:79,650,711, *p* = 2.1 × 10^–6^; *TRPM3*, rs760461668, *p* = 5 × 10^–8^; *SPTY2D1,* rs756851199, *p* = 1.6 × 10^–8^; and *TSPO,* rs566547284, *p* = 2.4 × 10^–6^). *PHIL* encoded protein is involved in pancreatic β-cell proliferation and survival, and TRPM3 protein mediates calcium signaling in pancreatic β-cells in response to glucose. A genetic risk score combining increasing insulin risk alleles of these four genes was associated with 53% (95% confidence interval 1.09, 2.15) increased odds of incident diabetes and 83% (95% confidence interval 1.35, 2.48) increased odds of impaired fasting glucose at follow-up. Our study uncovered novel gene-trait associations through the study of protein-coding variants and demonstrates the advantages of association screenings targeting diverse and high-risk populations to study variants absent in publicly available repositories.

## Introduction

Recent large-scale whole exome sequencing (WES) studies have identified loss of function (LOF) mutations (frameshift, splice donor, splice acceptor, and stop-gain variants) occurring at low allele frequency (< 1%) in populations^1,2^. These LOF mutations are predicted to inactivate or severely attenuate protein function and, therefore, provide a unique opportunity to assess their effects in humans. It is estimated that 3.5% of individuals harbor pathogenic or likely pathogenic variants that meet criteria for clinical action^3^. In addition, several of the genes considered LOF intolerant have no known human disease phenotype^2^.

There has been a great interest in phenotyping individuals with predicted protein-altering function altering variants (rare LOF and missense variants) to understand their health effects in populations. By linking WES data to electronic medical records of patients from a large health care organization, the DiscovEHR study identified novel associations of heterozygous LOF variants in *CSF2RB* with blood cell counts (basophil and eosinophil), LOF variants in *EGLN1* associated with hematocrit and hemoglobin, and deleterious missense variants in *G6PC* associated with triglyceride levels^4^. Studies in ancestrally distinct populations have also shown that a 2-step strategy that combines sequencing data of a subset of samples with subsequent genotyping in a large cohort can be an optimal way to maximize power while retaining experimental costs^5,6^. Low coverage sequencing has been shown to uncover novel variants in less studied populations^7^.

American Indians have a high burden of cardiometabolic diseases and may harbor rare coding variants that contribute to this risk. Building upon our ongoing investigation of exonic variation in American Indians using WES, we recently genotyped WES-identified single nucleotide variants (SNV) and small insertions/deletions (indels) with predicted protein-altering function in 1,127 Strong Heart Family Study (SHFS) participants. Approximately 43% of these genotyped variants are currently not present in publicly available repositories and are likely specific to American Indians. The goal of this study is to assess the clinical and biomarker phenotypic associations for carriers of these SNVs in American Indians. The identification of genes for specific phenotypes may provide insights into disease mechanisms and this knowledge could be applied to overall human populations including American Indians.

## Material and methods

### Population and phenotypes

The Strong Heart Study (SHS) is a population-based study of cardiovascular disease in American Indians recruited from tribes in Arizona, Oklahoma, and South and North Dakota^8^. The SHFS is a family component of the SHS, which examined 3776 members in 94 multigenerational families^9^. The first SHFS full family exam (2001-5, SHS Phase 4, baseline visit for this study) consisted of a personal interview, a physical exam and laboratory tests. A re-exam from 2006 to 2010 (Phase 5) had > 91% retention rate and measures were similar to the first exam. During the clinical visits, various categories of phenotypes were obtained including standardized physical measures (anthropometrics, blood pressure) and clinical data (diabetes, hypertension, medication use). A 12-h fasting serum and a spot urine sample were collected for laboratory biomarkers (complete blood cell count, serum lipids, liver and kidney function serum biomarkers, and metabolic biomarkers such glucose, insulin and HbA1c). DNA was extracted for genetic studies^8^. Pedigree relationships and identity-by-descent (IBD) sharing were estimated as previously described^10^. One tribe withdrew its consent to participate in future investigations and was not included in this analysis. The study was approved by the institutional review boards (IRBs) at each field center, and all participants gave written informed consent.

This study used existing data from a case–control study of chronic kidney disease (*n* = 555) and controls (*n* = 572) which included SHFS participants from two clinical centers (Oklahoma and the Dakotas) selected from 24 pedigrees. Cases were defined by a self-reported kidney failure (dialysis or transplant, *n* = 28), an eGFR less than 60 ml/min/1.73 m^2^ (*n* = 233) and/or urine albumin to creatinine ratio (UACR) ≥ 30 mg/g in any of the two clinical visits (*n* = 322, including *n* = 123 with both low eGFR and high UACR). Individuals without chronic kidney disease at two clinical visits and age > 40 years were selected as controls based on an eGFR > 80 ml/min/1.73 m^2^, and an UACR < 30 mg/g.

Phenotype definitions are shown in Table S1. Briefly, for participants using anti-hypertensive medications, we added 15 and 10 mmHg to their measured systolic and diastolic blood pressures, respectively. Estimated glomerular filtration rate (eGFR) was calculated using the serum creatinine-based Chronic Kidney Disease Epidemiology Consortium equation. LDL cholesterol (LDL-C) was estimated by the Friedewald formula for samples with triglycerides < 400 mg/dl and individuals were not taken statins at the time of lipid measures. For analyses of fasting glucose and insulin, we excluded individuals with diabetes. HOMA-IR (mmol/L) was calculated among non-diabetic individuals using the equation: fasting insulin in mU/L ^∗^ fasting glucose in mmol/L)/22.5^11^. Incident diabetes was defined by a new-onset fasting glucose > 126 mg/dL (7.0 mmol/L) and/or use of diabetic medications at follow-up. Incident impaired fasting glucose was defined by a new fasting glucose between 100 mg/dL (5.6 mmol/L) and 125 mg/dL (6.9 mmol/L) at follow-up. A reference group was selected of participants with fasting glucose < 100 mg/dL (5.6 mmol/L) at baseline and follow-up.

### Molecular data: low pass WES and Amerindian custom genotyping panel

SNVs/indels tested in this study were selected from a low coverage WES data of 94 distantly related SHFS participants, selected to maximize the diversity across founders to identify the genetic variability in this population (given lack of publicly available reference panels for American Indians). Participants for the WES were selected from pedigrees with large number of descendants and were not ascertained for a disease or trait. WES used Illumina TruSeq Custom Amplicon assay which captured > 200,000 exons in > 20,000 genes, resulting in ~ 62 Mb of targeted genomic regions, and high quality and genome coverage (mean call rate = 0.98, mean transition-transversion = 2.5, mean coverage at 10x = 80%).

We selected 2709 variants (SNVs/indels) for genotyping through an Illumina custom panel. Criteria for variant selection were: (1) observed in at least two individuals, (2) not present in publicly available databases (dbSNP, Exome Sequencing Project [ESP], 1000 Genomes Project) at the time of variant selection (2015), and (3) predictive functionality based on Genome Variant Server (frameshift, splice-3, splice-5, stop-gain of function, stop-loss of function, and missense variants). We also included some variants within 3’ or 5’ UTR or introns to complete the custom iSelect Illumina panel. Among variants genotyped, 144 failed manufacturing, 1357 were homozygous in our samples, and two were excluded due to call rates < 95%. The final sample for this study included 1127 individuals and 1206 SNVs/indels, and there was no overlap of participants with WES and those genotyped with the custom Illumina panel.

### Annotation of SNVs/indels

We performed extensive annotation of all exonic variants (both SNVs and indels) using the Whole Genome Sequencing Annotator (WGSA) based on reference hg19^12^. Variants were annotated as loss of function (essential splice sites, stop gain, stop lost, start lost, frameshift splice), non-synonymous, synonymous, and protein altering indels. This annotation also includes scores pertaining to functionality (Functional Analysis through Hidden Markov Models [FATHMM-MKL, http://fathmm.biocompute.org.uk/index.html]^13^, MetaSMV^14^, Combined Annotation Dependent Depletion [CADD]^15^, M-CAP^16^, likelihood ratio test (LRT)^17^), conservation (SIFT, Polyphen2), population allele frequencies (1000 Genomes Project, Exome Aggregation Consortium [ExAC], gnomAD), and disease-related annotations (ClinVar). This annotation was used to assess the potential impact of the variants in protein function and to identify SNVs/indels that are novel, i.e., not present in the repositories listed above at the time of the annotation. We assigned variants as deleterious if there was an agreement among more than 3 different annotation tools as proposed by the American College of Medical Genetics (ACMG) for a supporting level of pathogenic classification by computational prediction for nonsynonymous and LOF variants^18^.

### Statistical analyses

The main goal of analyses was to identify gene-phenotype associations for exonic variants while accounting for the case–control sampling and confounders. Traits were preprocessed through inverse normal transformation or outlier removal as needed. For a trait without transformation, observations more than 5 standard deviations away from the sample mean were set as outliers, with their corresponding values set to missing. No outliers were removed for traits that underwent inverse-normal transformation. Large pedigrees were split into families with no more than 33 members, by copying a child of a family and his/her genotype but not using the phenotype data^19^, as required for analyses of large-pedigree data using Merlin^20^.

We performed linear mixed model association analyses for each SNV/indel to account for family relatedness, which was implemented using the Merlin software^19^. We used additive genetic models and adjusted for age, sex, case–control status, and the first 10 genetic principal components estimated from a genome-wide genotype panel. For analyses of fasting insulin and glucose, we additionally adjusted for body mass index (BMI), reported as insulin_adjBMI_ and glucose_adjBMI_. Only variants with a minimum minor allele count (MAC) of 10 were tested in association analyses.

Given the genetic correlation among SNVs/indels due to linkage disequilibrium, we used a p-value threshold for significance of < 5.5 × 10^–6^, which accounts for 9,122 effective independent tests. The number of tests was calculated based on the extended Simes method^21^, part of the GATES method to calculate the effective number of independent tests^22^.

In secondary analyses, we combined genotypes of four insulin_adjBMI_-related SNVs using an unweighted genetic risk score that sums the fasting insulin_adjBMI_ increasing risk alleles for each participant and tested their association with incident diabetes and impaired fasting glucose at follow-up visit (Phase 5).

### Validation of associations

Replication was assessed in two cohorts of American Indians living in Arizona who had undergone WES performed by Regeneron Genetics Center (Tarrytown, New York). One cohort with WES data is part of a community-based study of Pima Indians (*N* = 6809) and the other cohort represents Urban Indians living in Phoenix Arizona (*N* = 850). Some variants were either not identified or had < 10 copies of the alternative allele in replication studies. Serum creatinine was not available in the replication cohorts. Therefore, two variants were tested for replication: rs779392624 for triglycerides and rs760461668 for fasting insulin_adjBMI_.

We also performed look ups for variants using publicly available data from the Type 2 Diabetes Knowledge Portal (https://t2d.hugeamp.org/), which included two pre-print whole genome sequencing (WGS) publications from the Trans-Omics for Precision Medicine (TOPMed) project on fasting insulin_adjBMI_ and Type 2 diabetes, respectively^23,24^, and the Metabolic Diseases Knowledge Portal (https://hugeamp.org/) for variants and genes related to our lipids and creatinine findings. Additional evidence for plausibility was obtained through experimental studies including genetic knockout animal studies.

### Ethnic statement

The study has been carried out in accordance with Declaration of Helsinki. The study was approved by the Institutional Review Boards of the participating Institutions (MedStar Research Institute, University of Oklahoma Health Science Center, Aberdeen Area IRB), and by the participating American Indian tribes^8,9^. All participants gave informed consent for genetic studies.

## Results

**Figure 1:**
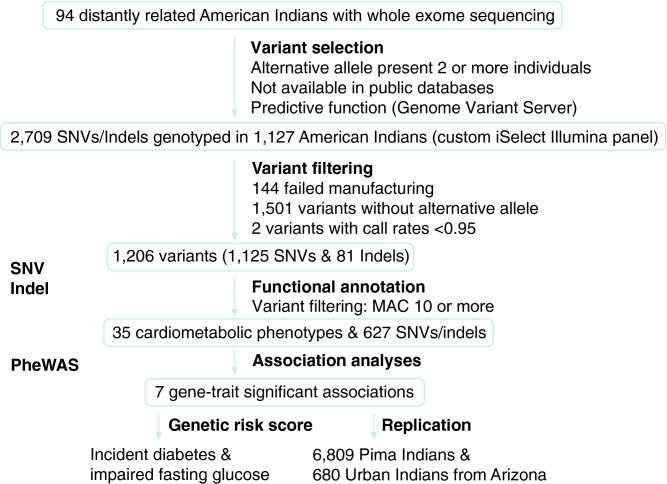
Study design for discovery, replication and follow-up analyses.

The study design is shown in Fig. 1, clinical and biomarker phenotypes in Table S1, and participant characteristics in Table S2.

### Functional annotation of variants and Amerindian-specific (novel) variants

Among 1206 genotyped variants that passed quality control (1125 SNVs and 81 indels within 1079 genes), 1162 were exonic SNVs/indels, and 44 were located in introns, 3’ or 5’ UTR (Table S3). Among exonic SNVs/indels 1024 were missense variants, 97 were frameshift substitutions, 20 were stop-gain or stop-loss variants, 20 were splice donor/acceptor and 1 was a synonymous variant. Most of the indels were frameshifts (*n* = 67, 83%) or splice donor/acceptors (*n* = 5, 6%). Of missense SNVs/indels, 114 (11%) were predicted damaging by MetaSVM and 641 (63%) by FATHMM-MKL annotations. Most variants were low frequency or rare (*n* = 85 singleton, *n* = 579 had a MAC < 10). The mean MAC was 20 (range of 1–1064).

By querying the genotyped SNVs/indels in publicly available databases, 518 SNVs/indels were not present in dbSNP, including 339 variants also not present in gnomAD exome as per June/2019. These variants were considered novel. Most of the SNVs present in gnomAD had higher allele frequency in our sample compared to other populations (Figure S1). The annotation of novel SNVs was missense SNVs/indels (*n* = 228), frameshift substitutions (*n* = 73), stop-gain or stop-loss variants (*n* = 8), and splice acceptor/donor (*n* = 11). All genotyped indels were novel. Of novel missense SNVs/indels, 44 (12%) were predicted deleterious by MetaSVM and 252 (66%) by FATHMM-MKL. All variants predicted deleterious by MetaSVM were also predicted deleterious by FATHMM-MKL (Table S3). In summary, our genotyped exome variants are composed of mostly low frequency and rare variants, likely American Indian-specific and enriched for predicted functionality.

### Association results with 35 phenotypes

Table 1 summarizes the main findings for variants reaching the significance threshold for at least one trait for adjusted models. Results for all variants (with a MAC ≥ 10, *n* = 627 variants) are shown in Table S4. Two novel SNVs at *EXTL2* (chr1:101,342,412, MAC = 33, *p* = 8.7 × 10^–9^) and *ACACA* (chr17:35,518,712, MAC 26, *p* = 2.9 × 10^–7^) were associated with low serum creatinine. The *EXTL2* SNV explained 2.42% of serum creatinine variance. This variant was associated with higher eGFR (*p* = 4.2 × 10^–4^) and lower uric acid (*p* = 0.03) among overall participants, although findings did not reach the multiple-testing significance threshold. The SNV at *ACACA* explained 1.87% of serum creatinine variance and was nominally associated with increased eGFR (*p* = 0.02).

**Table 1:** Main association results for variant-trait significant findings.

Trait	Chr:position (hg19)	Gene	Marker exonic function	Amino acid change	Coded/Other allele	Minor Allele Count	N	Effect	Variance explained (%)	*p*-value	Functional prediction of SNV*
Serum creatinine	1:101,342,412	*EXTL2*	Missense	p.M148V	G/A	33	1125	− 0.527	2.42	8.7 × 10^–9^	Deleterious
Serum creatinine	17:35,518,712	*ACACA*	Missense	p.P1683S	A/G	26	1125	− 0.506	1.87	2.9 × 10^–7^	Deleterious
Fasting triglycerides	17:67,149,477	*ABCA10*	rs779392624 missense	p.G1369W	A/C	17	1124	− 0.696	2.15	7.5 × 10^–9^	Deleterious
Fasting insulin	6:79,650,711	*PHIP*	Missense	p.T1722I	A/G	28	790	− 0.369	1.30	2.1 × 10^–6^	Deleterious
Fasting insulin	9:73,152,248	*TRPM3*	rs760461668 missense	p.V1086M	A/G	185	792	0.166	1.70	4.8 × 10^–8^	Deleterious
Fasting insulin	11:18,637,366	*SPTY2D1*	rs756851199 missense	p.V152A	G/A	109	793	0.220	1.75	1.6 × 10^–8^	Neutral/tolerant
Fasting insulin	22:43,557,062	*TSPO*	rs566547284 missense	p.G63S	A/G	26	793	0.396	1.45	2.4 × 10^–6^	Deleterious

For nonsynonymous rare variants and LOF variants, functional prediction algorithms were used to classify a SNV as deleterious based on agreement for at least three algorithms of prediction methods (see methods and Table S3). All SNVs listed in Table 1 had a CADD Phred score > 10–20, which is considered deleterious, except for rs756851199. Models adjusted for age, sex, center, and the first 10 principal components of ancestry. Fasting insulin was tested among non-diabetic individuals in models additionally adjusted for BMI. Amino acid change provided by the Variant Effect Predictor tool. *N* total number of participants. N/A, not available. Note three SNVs are not present in a publicly available database and lack rs#. Significance threshold *p* = 4.9 × 10^–6^ is based on number of SNVs and phenotypes tested.

A missense SNV at *ABCA10* (rs779392624, MAC = 17, *p* = 7.5 × 10^–9^) was associated with lower fasting triglycerides levels, and it explained 2.15% of triglyceride variability in our data (Table 1). The SNV was not associated with HDL cholesterol (HDL-C, *p* = 0.32) or LDL-C (*p* = 1.00) (Table S4).

Four SNVs were associated with fasting insulin_adjBMI_ among non-diabetic individuals. A novel missense variant at *PHIP* (chr6:79,650,711, MAC = 28, *p* = 2.1 × 10^–6^) was associated with decreased fasting insulin and explained 1.30% of insulin variance. It was also nominally associated with reduced eGFR (*p* = 0.01) but not with fasting glucose. A missense SNV of *TRPM3* (rs760461668, MAC = 185, *p* = 4.8 × 10^–8^) was associated with increased fasting serum insulin and explained 1.70% of insulin variance. This SNV was nominally associated with lower serum albumin (*p* = 0.03), fibrin (*p* = 0.03), UACR (*p* = 0.03) and LDL-C (*p* = 0.02) and higher triglycerides (*p* = 3.4 × 10^–4^). A missense SNV at *SPTY2D1* (rs756851199, MAC = 109, *p* = 1.6 × 10^–8^) was associated with increased fasting insulin and 1.75% variance in serum insulin. A SNV at *TSPO* (rs566547284, MAC = 26, *p* = 2.4 × 10^–6^) was associated with increased fasting insulin and it explained 1.45% of the variance of serum insulin.

### SNV and gene validation

Two variants which had ≥ 10 carriers in each of two cohorts of southwestern American Indians were analyzed for replication (rs779392624 for triglycerides and rs760461668 for fasting insulin), but the associations for these rare variants were not significant (Table S5). Two SNVs were available in the Trans-Omics for Precision Medicine (TOPMed) WGS summary statistics for fasting insulin_adjBMI_ (*n* = 23,211) and type 2 diabetes (*n* = 29,794). rs756851199 (*SPTY2D1)* was significantly associated with fasting insulin_adjBMI_ (*p* = 0.001) but rs760461668 (*TRPM3*) was not associated with insulin_adjBMI_ (*p* = 0.10).

Given these SNVs were rare or not available in published studies, we examined the evidence for association of any SNV within the identified genes for our traits. A gene-level analysis reported in the Metabolic Diseases Knowledge Portal showed associations at *ABCA10* with triglycerides, *PHIP* with fasting insulin_adjBMI_ and type 2 diabetes, and *SPTY2D1* with type 2 diabetes (Table S6). The lowest p-value associations for SNVs in the TOPMed WGS studies of fasting insulin_adjBMI_ and type 2 diabetes for SNVs for our gene-traits were *SPTY2D1 (p* = 2 × 10^–6^), *PHIP* (*p* = 2 × 10^–4^), *TSPO* (*p* = 8 × 10^–4^) and *TRPM3 (p* = 8 × 10^–7^) for fasting insulin_adjBMI_, and *TRMP3* (*p* = 2 × 10^–3^) for type 2 diabetes (Table S6).

### Insulin-based genetic risk score and incident diabetes and impaired fasting glucose

Using an unweighted genetic risk score, we examined patterns of associations for the four insulin-related SNVs in relation to development of type 2 diabetes and impaired fasting glucose at follow-up. Individuals carried 0 to 4 insulin-increasing risk alleles from *PHIL, TRPM3, SPTY2D1* and *TSPO.* Incident diabetes and impaired fasting glucose were obtained from a mean 5.3 years (standard deviation 1.1) from SHFS baseline visit. Among participants with normal fasting glucose at baseline, each added risk allele was associated with 53% odds of developing diabetes (*p* = 0.015) and 83% odds of developing impaired fasting glucose (*p* < 0.0001) at follow-up in models adjusted for age, sex, center, case-control status and principal components (Table 2). The association with incident diabetes was attenuated with further adjustment for BMI (*p* = 0.05), but the association with incident impaired fasting glucose was unchanged by BMI adjustments (*p* = 0.0001). The genetic risk score was strongly associated with increased log-transformed HOMA-IR at baseline visit among participants without diabetes in models adjusted for age, sex and case-control status (*N* = 793, *p* < 0.001).

**Table 2:** Association of insulin-related SNV genetic risk score with incident diabetes and impaired fasting glucose.

Incident outcomes	*N* cases/*N* total	Odds ratio (95% C.I.) Model 1	Odds ratio (95% C.I.) Model 2
Diabetes	103/571	1.53 (1.09, 2.15)	1.45 (0.997, 2.10)
Impaired fasting glucose	161/609	1.83 (1.35, 2.48)	1.84 (1.35, 2.49)

All analyses are adjusted for age, sex, center, case-control status, principal components (Model 1) and additional adjustments for BMI (Model 2). *C.I.* confidence interval, *N* number. All outcomes were obtained at follow-up visit. Genetic risk score was calculated by the unweighted sum of increasing insulin risk alleles of the SNVs (chr6:79,650,711, rs760461668, rs756851199, rs566547284).

## Discussion

This study identified associations of several predicted deleterious rare and low frequency exonic variants with cardiometabolic biomarkers and clinical traits in American Indians. These findings include seven gene-trait significant associations for lipids, glucose/insulin and kidney traits. Several genes identified have not been previously reported in genome-wide association studies for these traits, although the evidence for their biological function is supported by experimental studies (Table 3). For example, the *ABCA10* gene identified in association with lower fasting triglycerides is a cholesterol-responsive gene and encoded protein is involved in macrophage lipid homeostasis. Two recent studies have reported association of variants at the *ABCA10* loci with lipid traits including an intergenic variant **(**rs12453914) associated with triglycerides (*p* = 1.67 × 10^–6^) although findings were not genome-wide significant^23,24^. Genes identified in this study could be prioritized to uncover functional rare variants for these cardiometabolic traits.

**Table 3:** Supporting evidence for genes and associated traits. For replication of gene-trait associations, see Table S6.

Gene	Trait	Known function	Relation to associated trait
*EXTL2*	Serum creatinine	The gene activity relates to regulation of heparan sulfate biosynthesis^39^. Heparan sulphate proteoglycans interact with proteins and influence a variety of cellular and developmental processes^40^	Heparan sulfate are major components of the glomerular filtration barrier in kidneys
*ABCA10*	Triglycerides	Member of ABCA6-like transporters. ABCA10 protein is involved in macrophage lipid homeostasis and its expression is suppressed by cholesterol import into macrophages^41^	Other ABCA transporters have known physiological function in transmembrane transport of endogenous lipid substrates. For example, ABCA1 regulates high-density lipoprotein metabolism
*PHIP*	Fasting insulin (adjusted for BMI)	The encoded protein selectively interacts with the IRS-1, and IRS-1 has a central role in the downstream effects of insulin and insulin-like growth factor-1^42^	PHIP controls β-cell proliferation and survival^31^. *Phip* mutant mice have postnatal growth deficit and develop hypoglycemia^32^. *PHIP* rare SNVs associated with childhood obesity, insulin resistance and repression of pro-opio melanocortin^38^
*TRPM3*	Fasting insulin (adjusted for BMI)	Transient receptor potential melastatin 3 (TRPM3) channels are non-selective cation channels that are expressed in insulinoma cells and pancreatic β-cells, and are important for cellular calcium signaling and homeostasis. TRPM3 mediates calcium signaling in pancreatic β-cells in response to glucose stimuli, supporting its role in pancreatic β-cells function^25,28^	*Trpm3*-deficient mice do not show alterations in resting blood glucose levels in agreement with our findings^43^. TRPM3 is a target for the PPARgamma agonist anti-diabetic drugs
*TSPO*	Fasting insulin (adjusted for BMI)	Translocator protein (TSPO) is a high-affinity cholesterol- and drug-binding mitochondrial protein	*Tspo* gene conditional knockout mice have shown a lack of response to adrenocorticotropic hormone and sustained hyperglycemia, which suggest a pre-diabetes phenotype^33^

Among the four genes identified for fasting insulin among non-diabetic individuals, *TRPM3* is expressed in insulinoma and pancreatic β-cells, and the protein is involved in calcium signaling in pancreatic β-cells in response to glucose stimuli^25–28^. TRPM3 channel activation has been shown to be inhibited by thiazolidinediones antidiabetic drugs such as pioglitazone and troglitazone, which are peroxisome proliferator-activated receptor (PPAR)gamma agonists^29,30^. *PHIP* encodes a protein that interacts with insulin receptor substrate 1 (IRS-1) and is involved in β-cell proliferation and survival^31^. Mice lacking *PHIP* develop hypoglycemia^32^. *TSPO* is involved in mitochondrial cellular metabolism and conditional *tspo* knockout mice manifest chronic hyperglycemia^33^. We were able to replicate the *SPTY2D1* SNV for fasting insulin_adjBMI_ in the TOPMed data, but not the other SNVs which were rare or not present in datasets. However, we identified some evidence to support associations for our gene-traits through look-ups of gene-based findings and low p-value SNVs for our traits within the identified genes.

Among the four genes (*PHIP*, *TRPM3*, *SPTY2D1* and *TSPO*) associated with fasting insulin and combined into a genetic risk score, we showed that carriers of insulin increasing risk alleles had higher odds of developing diabetes and impaired fasting glucose at follow-up. These findings and the association of the genetic risk score with the HOMA-IR support insulin resistance as a mechanism for development of diabetes and impaired fasting glucose in our population. The attenuation of the association of the genetic risk score with incident diabetes when adjusting for BMI suggests mediation by obesity. In a randomized clinical trial, the PPARgamma agonist pioglitazone has shown to reduce the risk of diabetes among individuals with impaired glucose tolerance^34^. American Indians have a high prevalence of both type 2 diabetes and impaired glucose tolerance, and one could speculate that carriers of the *TRPM3* SNV may benefit from using preferentially these medications for diabetes prevention. Therefore, the study of exonic variants can uncover not only biological relationships for gene-traits not previously reported in genome-wide association studies but also provide potential clinical applications for gene findings in high-risk populations such as ours.

An important aspect of this project is the study of American Indian-specific variants. We have shown that 1/3 of the variants assessed in this study are still not available in repositories. This includes all identified indels. The remaining variants are found in low frequency in Hispanics in the gnomAD exome variant database, given some Hispanics have Amerindian admixture^35^. These variants are not included in commercial GWAS genotyping panels. Therefore, they have not been previously queried for disease risk in large consortia of complex traits. The low coverage WES used to identify Amerindian SNVs has the advantage of low cost and capturing most of low frequency/common variants in our data, given variant reference panels are not available for our population. Studies have shown that low coverage WES identified variants perform well in association studies without an excess of false positive, although this strategy may have missed some variants^36,37^. This study exemplifies a major advantage of leveraging WES findings to select predicted functional variants for association screenings in genetically less well-characterized populations.

Our strategy for variant selection was driven by the current lack of reference panels for American Indians. We selected predicted functional variants from a WES performed in a subset of American Indian participants of the SHFS. We focused on variants that were not present in public repositories at the time of genotyping and then built a customized panel to genotype them in a larger sample of American Indians. This strategy offered some challenges including a large number of variants without an alternative allele (40%) at genotyping due to their low frequency in the studied population, and limitations for replication of variants. The genetic risk score results likely overestimated the effect as they were applied to the discovery sample. American Indians are characterized by distinct cultural and linguistic features, and separated by large geographic distances allowing for genetic variation between groups to have occurred. Our study included American Indians from tribes in the Dakotas and Oklahoma, but not Southwestern tribes who were used for replication. Given these challenges, we believe that the best approach to validate our findings will be the functional characterization of our variants in experimental models, and future target search for LOF exonic variants in the genes that we identified in this study.

In support to this strategy and a potential role of *PHIP* in insulin resistance, a recent study identified an excess of very rare predicted deleterious variants at *PHIP* in childhood severe obese individuals compared to controls, with some *PHIP* carriers showing insulin resistance and early type 2 diabetes^38^. Functional in vitro experiments supported a role of *PHIP* in human energy homeostasis through transcriptional regulation of central melanocortin signaling pathways^38^. Our participants carrying the *PHIP* SNV A allele had similar BMI than non-carriers (29.2 [standard deviation 5.5] and 31.4 [6.7] kg/m^2^ for genotypes AG and GG, respectively, *p* = 0.18) and all analyses were adjusted for BMI.

In summary, this study of predicted functional Amerindian-specific exome variants identified seven gene-trait associations and uncovered potential new biological mechanisms and clinical implications for genes not previously reported to be associated with cardiometabolic traits. Our results add to the literature of exonic variants associated with cardiometabolic traits in American Indians.

## Supplementary Information

Supplementary Information 1.

Supplementary Information 2.

## Data Availability

The Strong Heart Study and the SHFS^9^ data is available through dbGaP Study Accession: phs000580.v1.p1 and upon request from the https://strongheartstudy.org/. The summary data are included in the online supplemental files.

## References

[1] MacArthur DG (2012). A systematic survey of loss-of-function variants in human protein-coding genes. Science.

[2] Lek M (2016). Analysis of protein-coding genetic variation in 60,706 humans. Nature.

[3] Abul-Husn NS (2016). Genetic identification of familial hypercholesterolemia within a single US health care system. Science.

[4] Dewey FE (2016). Distribution and clinical impact of functional variants in 50,726 whole-exome sequences from the DiscovEHR study. Science.

[5] Holm H (2011). A rare variant in MYH6 is associated with high risk of sick sinus syndrome. Nat. Genet..

[6] Sanna S (2011). Fine mapping of five loci associated with low-density lipoprotein cholesterol detects variants that double the explained heritability. PLoS Genet.

[7] Martin AR (2021). Low-coverage sequencing cost-effectively detects known and novel variation in underrepresented populations. Am. J. Hum. Genet..

[8] Lee ET (1990). The strong heart study. A study of cardiovascular disease in American Indians: design and methods. Am. J. Epidemiol..

[9] North KE (2003). Genetic and environmental contributions to cardiovascular disease risk in American Indians: the strong heart family study. Am. J. Epidemiol..

[10] Franceschini N (2006). A quantitative trait loci-specific gene-by-sex interaction on systolic blood pressure among American Indians: the Strong Heart Family Study. Hypertension.

[11] Matthews DR (1985). Homeostasis model assessment: insulin resistance and beta-cell function from fasting plasma glucose and insulin concentrations in man. Diabetologia.

[12] Liu X (2016). WGSA: an annotation pipeline for human genome sequencing studies. J. Med. Genet..

[13] Shihab HA (2014). Ranking non-synonymous single nucleotide polymorphisms based on disease concepts. Hum. Genomics.

[14] Dong C (2015). Comparison and integration of deleteriousness prediction methods for nonsynonymous SNVs in whole exome sequencing studies. Hum. Mol. Genet..

[15] Rentzsch P, Witten D, Cooper GM, Shendure J, Kircher M (2019). CADD: predicting the deleteriousness of variants throughout the human genome. Nucl. Acids Res..

[16] Jagadeesh KA (2016). M-CAP eliminates a majority of variants of uncertain significance in clinical exomes at high sensitivity. Nat. Genet..

[17] Chun S, Fay JC (2009). Identification of deleterious mutations within three human genomes. Genome Res..

[18] Richards S (2015). Standards and guidelines for the interpretation of sequence variants: a joint consensus recommendation of the American College of Medical Genetics and Genomics and the Association for Molecular Pathology. Genet. Med..

[19] Abecasis GR, Cherny SS, Cookson WO, Cardon LR (2002). Merlin–rapid analysis of dense genetic maps using sparse gene flow trees. Nat. Genet..

[20] Scuteri A (2007). Genome-wide association scan shows genetic variants in the FTO gene are associated with obesity-related traits. PLoS Genet..

[21] Li MX, Gui HS, Kwan JS, Sham PC (2011). GATES: a rapid and powerful gene-based association test using extended Simes procedure. Am. J. Hum. Genet..

[22] Li MX, Yeung JM, Cherny SS, Sham PC (2012). Evaluating the effective numbers of independent tests and significant p-value thresholds in commercial genotyping arrays and public imputation reference datasets. Hum. Genet..

[23] Feitosa MF (2020). Gene discovery for high-density lipoprotein cholesterol level change over time in prospective family studies. Atherosclerosis.

[24] Lu X (2016). Genetic susceptibility to lipid levels and lipid change over time and risk of incident hyperlipidemia in Chinese populations. Circ. Cardiovasc. Genet..

[25] Thiel G, Muller I, Rossler OG (2013). Signal transduction via TRPM3 channels in pancreatic beta-cells. J Mol Endocrinol.

[26] Held K (2015). Activation of TRPM3 by a potent synthetic ligand reveals a role in peptide release. Proc. Natl. Acad. Sci. U. S. A..

[27] Wagner TF (2008). Transient receptor potential M3 channels are ionotropic steroid receptors in pancreatic beta cells. Nat. Cell Biol..

[28] Becker A (2020). Control of insulin release by transient receptor potential melastatin 3 (TRPM3) Ion channels. Cell Physiol. Biochem..

[29] Thiel G, Rubil S, Lesch A, Guethlein LA, Rossler OG (2017). Transient receptor potential TRPM3 channels: pharmacology, signaling, and biological functions. Pharmacol. Res..

[30] Majeed Y (2011). Rapid and contrasting effects of rosiglitazone on transient receptor potential TRPM3 and TRPC5 channels. Mol. Pharmacol..

[31] Podcheko A (2007). Identification of a WD40 repeat-containing isoform of PHIP as a novel regulator of beta-cell growth and survival. Mol. Cell Biol..

[32] Li S (2010). The full-length isoform of the mouse pleckstrin homology domain-interacting protein (PHIP) is required for postnatal growth. FEBS Lett..

[33] Fan J, Campioli E, Papadopoulos V (1865). Nr5a1-Cre-mediated Tspo conditional knockout mice with low growth rate and prediabetes symptoms - a mouse model of stress diabetes. Biochim. Biophys. Acta Mol. Basis Dis..

[34] DeFronzo RA (2011). Pioglitazone for diabetes prevention in impaired glucose tolerance. N. Engl. J. Med..

[35] Conomos MP (2016). Genetic diversity and association studies in us hispanic/latino populations: applications in the hispanic community health study/study of Latinos. Am. J. Hum. Genet..

[36] Pasaniuc B (2012). Extremely low-coverage sequencing and imputation increases power for genome-wide association studies. Nat. Genet..

[37] Gilly A (2019). Very low-depth whole-genome sequencing in complex trait association studies. Bioinformatics.

[38] Marenne G (2020). Exome sequencing identifies genes and gene sets contributing to severe childhood obesity, linking PHIP variants to repressed POMC transcription. Cell Metab..

[39] Katta K (2015). Reduced expression of EXTL2, a member of the exostosin (EXT) family of glycosyltransferases, in human embryonic kidney 293 cells results in longer heparan sulfate chains. J. Biol. Chem..

[40] Nadanaka S, Kitagawa H (1862). Exostosin-like 2 regulates FGF2 signaling by controlling the endocytosis of FGF2. Biochim. Biophys. Acta Gen. Subj..

[41] Wenzel JJ (2003). ABCA10, a novel cholesterol-regulated ABCA6-like ABC transporter. Biochem. Biophys. Res. Commun..

[42] Farhang-Fallah J, Yin X, Trentin G, Cheng AM, Rozakis-Adcock M (2000). Cloning and characterization of PHIP, a novel insulin receptor substrate-1 pleckstrin homology domain interacting protein. J. Biol. Chem..

[43] Vriens J (2011). TRPM3 is a nociceptor channel involved in the detection of noxious heat. Neuron.

