# Supplementary material for: Association of protein function-altering variants with cardiometabolic traits: the strong heart study
Source: Sci Rep. 2022 Jun 4;12:9317. doi: 10.1038/s41598-022-12866-2 (PMC9167281; doi:10.1038/s41598-022-12866-2)
Supplement: Supplementary file 1 — Supplementary Information 1. [file 41598_2022_12866_MOESM1_ESM.pdf]

Supplemental Material- Figures

Association of protein function-altering variants with cardiometabolic traits: the Strong Heart Study

Yue Shan, Shelley A Cole, Karin Haack, Phillip E Melton, Lyle G Best, Christopher Bizon, Sayuko Kobes, Çiğdem Köroğlu, Leslie J Baier, Robert L Hanson, Serena Sanna, Yun Li, Nora Franceschini

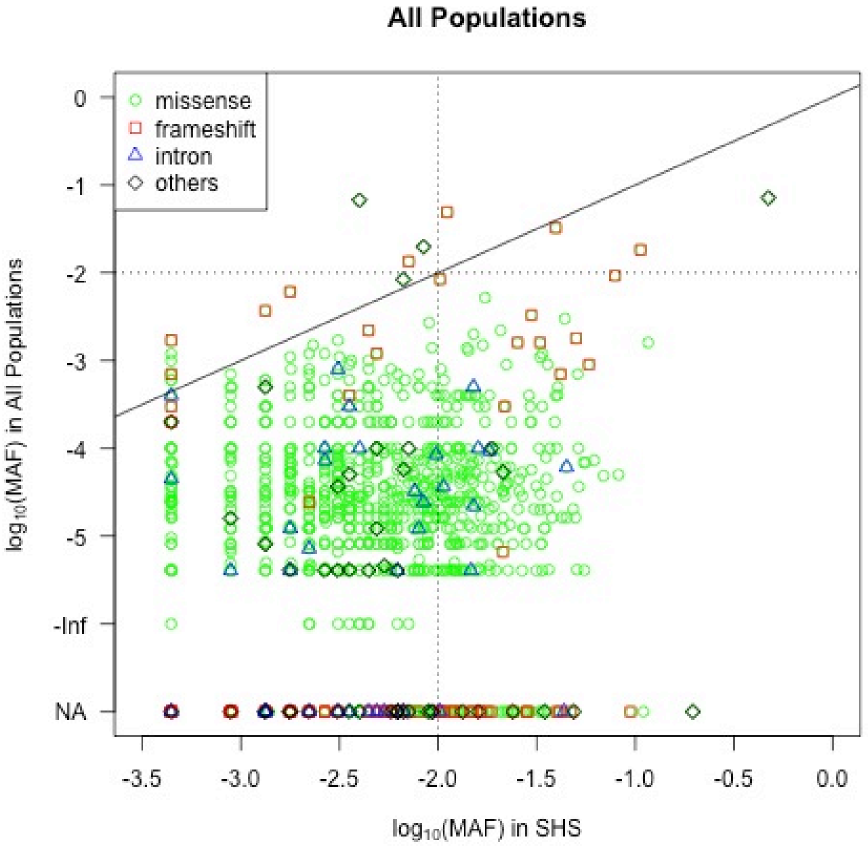
All Populations
missense
frameshift
intron
others
log10(MAF) in All Populations
log10(MAF) in SHS
0
-1
-2
-3
-4
-5
-Inf
NA
-3.5
-3.0
-2.5
-2.0
-1.5
-1.0
-0.5
0.0

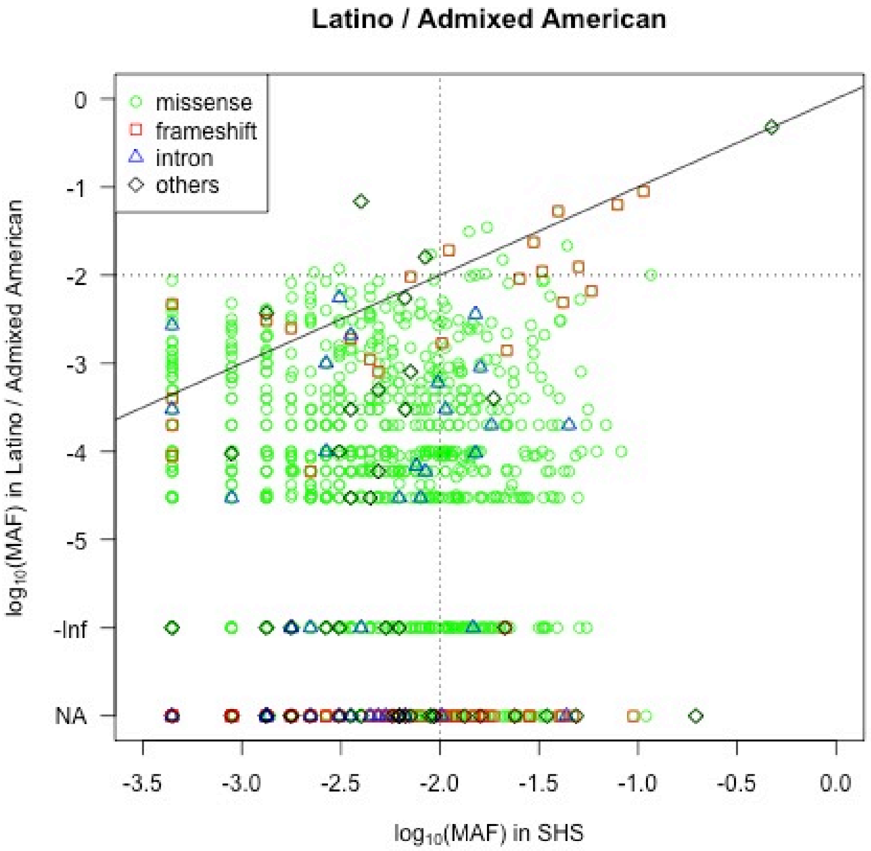
Latino / Admixed American
missense
frameshift
intron
others
log10(MAF) in Latino / Admixed American
log10(MAF) in SHS
0
-1
-2
-3
-4
-5
-Inf
NA
-3.5
-3.0
-2.5
-2.0
-1.5
-1.0
-0.5
0.0

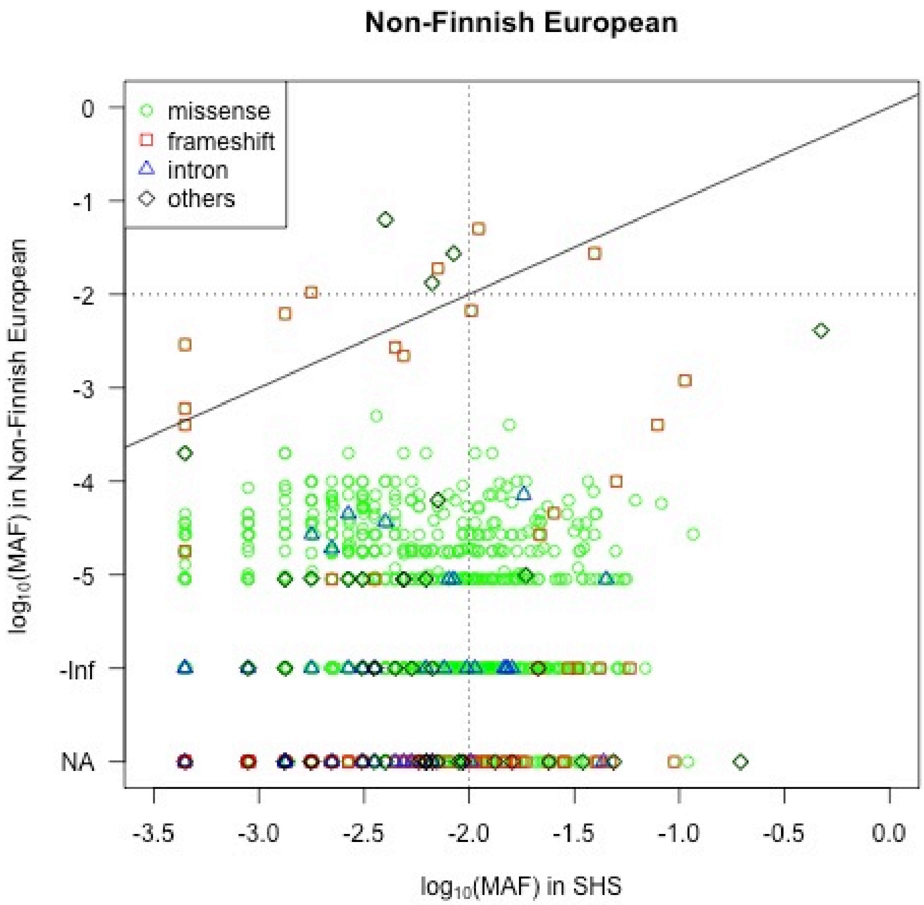

Figure S1. Comparison of allele frequency of exonic SNVs and indels genotyped in the SHFS and ancestrally different populations using gnomAD exome data. Each point is a SNV/indel plotted against their log10 minor allele frequency (MAF) in the SHFS (x-axis) and ancestrally different populations (y-axis). The solid line represents the 45-degree line passing the origin (0,0). Each dot on the line means the MAF are the same between SHFS and the compared population, dots below the line mean that the MAF in SHFS is larger than comparable population, dots above the line mean that the MAF in the SHFS is lower than comparable population. The functional annotation of variants (color and shape of the dots) are also provided. Note that some variants are currently not present in ancestrally different populations (Not available, NA).
